# Identifying communication barriers between nurses and patients from the perspective of Iranian nurses: a Q-methodology-based study

**DOI:** 10.1186/s12912-024-02136-z

**Published:** 2024-07-05

**Authors:** Reza Ghanei Gheshlagh, Syede Mona Nemati, Reza Negarandeh, Fatemeh Bahramnezhad, Pershang Sharifi Saqqezi, Hassan Mahmoodi

**Affiliations:** 1 Social Determinants of Health Research Center Research Institute for Health Development, Kurdistan University of Medical Sciences, Sanandaj, Iran; 2 Faculty of Nursing and Midwifery, Kurdistan University of Medical Sciences, Sanandaj, Iran; 3 Nursing and Midwifery Care Research Centre, School of Nursing and Midwifery, Tehran University of Medical Sciences, Tehran, Iran; 4 School of Nursing & Midwifery, Nursing and Midwifery Care Research Center, Tehran University of Medical Sciences, Tehran, Iran; 5 Department of Ear, Nose and Throat, kowsar Hospital, Kurdistan University of Medical Sciences, Sanandaj, Iran; 6 Vice Chancellor for Health Affairs, Health Education and Promotion Group, Kurdistan University of Medical Sciences, Sanandaj, Iran

**Keywords:** Communication, Nurse, Barriers, Patient, Q methodology, Iran

## Abstract

**Background:**

Effective communication is essential for high-quality healthcare, yet barriers often impede meaningful connection between nurses and patients. This study aimed to prioritize communication barriers between nurses and patients in Iranian hospitals, exploring nurses’ perspectives.

**Methods:**

Thirty-one nurses participated in a six-step Q methodology study to identify different perspectives on communication barriers. Participants sorted a set of statements based on their own experiences and beliefs.

**Results:**

The average age of participants was 38.07 (SD = 6.49), with 70% being women. Four distinct factors emerged, explaining 47% of the total variance in perspectives: Organizational factors and work conditions (20%), Emotional distress and psychological barriers (11%), Lack of mutual understanding and awareness (7%), and declining professional motivation and engagement among nurses (9%).

**Conclusion:**

These findings highlight the multi-faceted nature of communication barriers between nurses and patients in this context. Interventions should address organizational factors, emotional well-being of nurses, cultural awareness, and professional motivation to improve communication and ultimately, patient care. This study provides valuable insights for Iran and other developing countries that are facing similar challenges.

## Background

Effective communication lies at the heart of quality healthcare. Yet, the seemingly simple act of exchanging information between nurses and patients can be fraught with challenges, creating invisible walls that hinder understanding and optimal care [1]. Studies have demonstrated that the provision of quality patient care and recovery greatly depend on effective communication between healthcare providers and patients [2–4]. The Institute of Medicine (IOM), in its 2003 report on Health Professions Education, highlighted the importance of patient-centered care and emphasized that providing patient-centered care should be the cornerstone of healthcare professionals’ education [5].

Identifying the multi-faceted barriers to patient-centered care and communication in nurse-patient interactions, as highlighted in a recent literature review, paves the way for developing targeted interventions addressing institutional structures, communication training, environmental improvements, and personal/behavioral skills [6]. A cross-sectional qualitative study identified multiple barriers to effective therapeutic communication between nurses and patients, including: patient-related factors (sociodemographic attributes, rapport issues, misconceptions, language), nurse-related factors (workload, competence doubts, family interference, knowledge gaps, patient dissatisfaction, emotional reactions), and environment-related factors (unsuitable atmosphere, changes, noise) [7]. Patients in primary healthcare centers reported several communication barriers with nurses, including: understaffing, nurse disinterest, negativity, language barriers, nurse self-doubt, and workload stress [8].

A study in Iran highlighted the multifaceted nature of barriers to nurse-patient communication, categorized into four key areas: nurse-related factors (understaffing, negative attitude, language gaps, self-doubt), patient-related factors (underestimation of the impact of the communication, cultural differences, communication preferences), environmental factors (crowding, noise, privacy), and shared factors (mistrust, preconceived notions, cultural misunderstandings) [9]. While qualitative and quantitative studies have been conducted to identify barriers to nurse-patient communication from both nurses’ and patients’ perspectives, no mixed-methods study has yet been undertaken in this area [6–9]. A mixed-methods study employing Q methodology could offer a novel approach to understanding these barriers from the subjective viewpoints of healthcare professionals.

Q methodology is specifically designed to capture and analyze subjective viewpoints, which are often overlooked in traditional quantitative or qualitative research [10]. This allows for a deeper understanding of the personal perceptions, experiences, and beliefs that shape communication between nurses and patients. By applying Q methodology, researchers have gained valuable insights into the subjective experiences that contribute to challenges in health research, paving the way for more effective research practices [11, 12]. This study aimed to identify communication barriers between nurses and patients from the perspective of Iranian nurses using Q methodology.

## Method

The Q method, pioneered by William Stevens in the 1930s, unveils hidden perspectives by merging qualitative and quantitative approaches [13].

The study recruited 31 nurses who met the inclusion criteria and willingly participated. These nurses were employed at teaching hospitals affiliated with the Kurdistan University of Medical Sciences in Sanandaj, Iran. The study was approved by the Medical Ethics Board of Trustees (MEBoT) within the Tehran University of Medical Sciences (approval number: IR.TUMS.FNM.REC.1402.075). Written informed consent was obtained from all the participants after the first author briefed them on the study objectives. All the methods were employed per relevant guidelines and regulations (Declaration of Helsinki).

Q methodology, as outlined by Brown, involves six steps: defining the research topic (also known as the concourse, which establishes the area of interest to be explored), creating statements (the Q set, which represents diverse viewpoints within the concourse), selecting participants (the P set), conducting the sorting (Q sorting, which reveals the participants’ personal positions and relationships with the statements), and finally, analyzing and interpreting the sorting data to discern underlying patterns and perspectives [14].

### Step 1:

The term “concourse” refers to the collection of statements or phrases that represent the range of perspectives on a particular topic or issue. In this study, we developed a concourse, or Q sample, of 200 short statements using a two-phase hybrid approach. This approach combined scrutiny of research evidence with participant input.

Phase 1: We searched online databases like PubMed, Embase, Scopus, and Google Scholar using relevant Medical Subject Headings (MeSH) terms such as “communication barriers,” “nurses,” “patients,” “Iran,” and “hospital setting.” We focused on publications from 2015 to 2024. This search yielded 13 publications that reported barriers to nurse-patient communication, and these provided 99 short statements for the Q sample. Phase 2: We conducted semi-structured interviews with 31 nurses who had prior experience working with patients. We explored their perceptions and experiences regarding communication barriers between nurses and patients. This phase generated 101 short statements representing healthcare professionals’ perceived communication barriers. We conducted a one-on-one comparison of the statements retrieved from the literature review and the interviews. This process identified 52 short statements with enough overlap to be retained and move on to the next stage of the study.

### Step 2:

“Q set” refers to a carefully selected subset of statements or phrases drawn from the concourse. This subset represents the key aspects of the discourse space, enabling participants to meaningfully sort the statements based on their own perspectives. The Q set is typically composed of 40–60 statements, carefully chosen to represent a balance of viewpoints and to ensure the comprehensiveness of the discourse space [15]. In our study, we selected 52 statements out of 200 statements from the concourse.

### Step 3:

P-set refers to the sample of participants who engage in the Q sort exercise. This sample is carefully selected to represent the range of perspectives relevant to the research topic and the specific focus of the study. A P-set of 40–60 is found to be adequate for all purposes, chosen from a variety of backgrounds and experiences [16]. In this study, we selected 31 nurses as the P-set to represent the range of perspectives relevant to communication barriers with patients.

This study recruited nurses who had direct patient care experience in a hospital setting. Participants were also required to be willing and available to participate in the Q-sorting activity. Purposive sampling was employed to ensure a diverse P-set (participant set) reflecting a range of perspectives on communication barriers with patients. This involved collaborating with hospital administrators to identify and recruit nurses who met the inclusion criteria. This collaboration facilitated the identification and access to nurses who met the inclusion criteria. This approach ensured access to a relevant and representative sample of the target population.

### Data collection

Semi-structured interviews were conducted with nurses to gather additional perspectives and refine the concourse. Interviews were conducted at the participants’ workplaces for their convenience. At the beginning of each session, the study protocol was explained, and participants were encouraged to openly share their experiences without hesitation. We began the interviews with a general, open-ended question about their perceptions of communication barriers between nurses and patients in Iranian hospitals. These interviews provided in-depth insights from participants and helped ensure the relevance of the statements. In this study, we used the semi-structured interview. The guide to the topics of the interviews was:

1. Can you describe some of the main challenges you face when communicating with patients in the hospital?
2. Are there any specific situations where communication with patients is particularly difficult? (e.g., language barriers, anxious patients, cultural differences)
3. In your opinion, what factors related to the hospital environment or workload might hinder effective communication?
4. How do you typically overcome challenges in communication with patients?
5. Are there any specific communication strategies you find particularly helpful?
6. Does your hospital offer any training or resources to help nurses improve their communication skills?
7. How do communication barriers with patients impact the quality of care provided?
8. In your ideal world, what changes could be implemented within the hospital to improve communication between nurses and patients?
9. Is there anything else you would like to share about your experiences with communication and patient care?
10. Can you provide an example of a time when communication with a patient was particularly challenging?

#### Step 4:

Q sorting is the core of Q methodology, a research method that explores subjective perspectives and underlying patterns of thought within a discourse space. It involves presenting participants with a set of statements (the Q set) and asking them to sort the statements based on their own beliefs, attitudes, or opinions [17]. In our study, 31 nurses sorted 52 statements on a likert scale from + 5 to -5 based on their opinions.

To assess validity and reliability of the Q-sort and gather feedback from participants, the following methods were employed. Post-Sort Interviews: After completing the Q-sort, participants were invited to participate in brief interviews. These interviews allowed them to elaborate on their sorting decisions and provide feedback on the clarity and comprehensiveness of the statements. Participant Feedback Forms: Participants were also provided with feedback forms to anonymously share their thoughts on the Q-sort process, the statements, and the overall study experience. This feedback was valuable in identifying areas for improvement and ensuring the study was participant-centered.

#### Step 5 and 6:

**Analysis and Interpreting-** The data obtained from the Q sorts were entered into PQ-Method software version 2.35. The analysis and interpretation process was carried out in three stages: (a) factor identification, (b) conversion of factors into factor arrays, and (c) interpretation of factors using factor arrays.

A. **Factor Identification**: Factor extraction was performed in PQ-Method software using the following steps: (a) principal component analysis, (b) identification of hidden factors, (c) varimax rotation and evaluation of factor loadings for eigenvalues greater than 1.00, (d) calculation of the proportion of variance explained by the identified factors, and (e) differentiation of interpretable factors with at least two types of correlated Q [16].
B. **Conversion of Factors into Factor Arrays**: The observed correlation between each Q-sort and identified factors provides insight into the alignment between the Q-sorts and the identified factors [18]. This study utilized the manual marking mechanism in PQ-Method software, setting a minimum correlation coefficient of 0.357 as the cut-off point (the absolute value of the factor loading is greater than (2/58)/(√n), then the factor loading, respectively, was consider significant with 99% confidence if the value of n, which was equal to the number of phrases in the Q study (*n* = 52) was ordered for the identified factors [19]. The ordering of statements for each identified factor was determined based on these correlation coefficients. The order of statements in each factor is used to create the factor array for that factor. The factor array represents the ordering of that factor (perspective) and is determined using z-scores. In essence, the factor array determines for each factor at which level of the spectrum each statement lies; thus, a more accurate interpretation of each factor (mentality) can be achieved by examining the position of each statement. P-values were also determined from the z-scores to differentiate between statements, with a value of less than 0.05 considered significant compared to 0.01. [20].
C. **Factorial Interpretation Using Factor Arrays**: Distinct Q statements were identified, and each factor was interpreted in the context of its respective orderings. The defining statements for a particular factor were those with in the factor arrays with rank values of “+5”, “+4”, “-5”, and “-4”, and with distinct scores (*p* < 0.05). To confirm the recognition and interpretation of the factors among identified subgroups, post-P-set interviews were conducted after the Q-sorts to compare their scores in other factors.

## Results

The mean age of participants in this study was 38.07 (SD = 6.49). The majority of the participants were women (70%). On average, participants spent 19 min distributing items in the Q sorting process. (Table 1)

**Table 1:** Demographic participant’s characteristics

Variables	Frequency	Percent
**Gender**		
Male	12	38.7
Female	19	61.3
**Education level**		
Bachelor’s degree	20	64.5
Master’s degree	10	32.2
PhD degree	1	3.3
**Job duration**		
Less than 10	7	22.5
Between 10 to 20	19	61.3
More than 20	5	16.2

Four factors were extracted, explaining 47% of the total variance: Organizational factors and work conditions (20%), Emotional distress and psychological barriers (11%), Lack of mutual understanding and awareness (7%), and Declining professional motivation and engagement among nurses (9%). The rotated matrix of factors showed that the first factor was loaded by 19 nurses, the second by seven, the third by four, and the fourth by seven. After determining the factor scores in the rotated matrix, factor arrays were calculated. These arrays were used to form a Q table for each factor, assigning a score to each of the Q options. The Q options, identified for each factor, were arranged in order of importance (Table 2).

**Table 2:** The Q-set statements and factor arrays in the study of communication barriers between nurses and patients

Item	Statements	Factor 1	Factor 2	Factor 3	Factor 4
1	Lack of an adequate number of nurses in comparison to the high volume of patients.	**3	*0	*-3	*-2
2	Nurses’ reluctance to engage in effective communication with patients.	2	1	-1	0
3	Negative attitude displayed by nurses towards patients.	-2	-2	0	1
4	Language barrier between nurses and patients.	-4	-4	-5	*-3
5	Insufficient self-confidence among nurses.	-2	-2	0	-1
6	Cultural disparities between nurses and patients.	**-4	-3	-1	-3
7	Gender differences between nurses and patients.	-4	-4	-5	-5
8	Age gaps between nurses and patients.	-5	-5	-4	-4
9	Scarcity of time.	3	1	1	**-1
10	Inadequate understanding of the patient’s needs and condition.	-1	-1	0	*1
11	Patient’s lack of awareness regarding the role and responsibilities of nurses.	**1	**-1	5	5
12	Anxiety, pain, and physical discomfort experienced by patients.	-2	**4	-1	-1
13	Presence of critically ill patients in the department.	3	1	**-2	1
14	Overcrowded environment within the department.	1	1	**-1	**3
15	Inappropriate environmental conditions.	0	0	-2	0
16	Job dissatisfaction among nurses.	4	2	2	4
17	Uncontrolled presence of patients’ family members.	*1	2	3	**-4
18	Lack of trust in the competency of nurses.	-1	-3	-2	0
19	Insufficient cultural competence of nurses.	-3	*0	-2	-2
20	Nurses’ lack of responsibility in communicating with patients.	**-1	**-3	2	1
21	Excessive workload.	**5	3	2	**-3
22	Low salaries received by nurses.	**5	3	**-4	3
23	Religious differences between nurses and patients	-5	-5	**1	*-4
24	Inadequate provision of comfort facilities for nurses.	*4	0	*2	0
25	Lack of interest and motivation among nurses towards their profession.	3	**0	**-3	5
26	Nurses’ limited awareness of the concept of communication and communication skills	**2	-1	**4	-1
27	Nurses’ limited awareness of verbal and non-verbal behaviors.	2	0	1	-1
28	Inadequate understanding of patients’ needs and condition among nurses.	**-2	1	**-3	1
29	Negative experiences from previous interactions with patients.	0	*-1	2	0
30	Specific type of nursing work department.	-1	3	-2	2
31	Physical problems experienced by nurses.	0	**-4	-1	0
32	Lack of attention from nursing officials towards the communication between nurses and patients.	**1	-2	-1	**2
33	Insufficient training on communication principles provided to nurses.	**1	**-1	4	2
34	Patients’ lack of knowledge about the position and responsibilities of nurses	*-1	*2	**-3	*0
35	Negative attitude of patients towards nurses	2	1	*-1	1
36	Resistance and unwillingness of patients to engage in communication	0	0	0	1
37	Patients being in an unfamiliar hospital environment.	-2	**1	-2	-2
38	Nurses speaking rapidly and hurriedly	-3	-2	-4	-1
39	Usage of technical terms by nurses.	**-3	0	1	**-5
40	Incorrect interpretation of communication by nurses.	**0	**-2	3	3
41	Lack of trust, privacy, and confidentiality.	-3	-1	1	2
42	Mental well-being of nurses.	-1	-1	0	*-2
43	Low level of awareness among patients.	**0	2	4	3
44	A high number of shifts.	4	4	5	**-1
45	Inappropriate behavior of patients’ companions.	*1	**5	*0	*2
46	Limited experience of the nurse.	2	2	1	**-3
47	Absence of feedback from patients.	**-1	3	3	4
48	Inadequate supervision of nurses.	**0	**-3	3	4
49	Patients asking unnecessary questions.	-2	**5	**1	-2
50	Nurses’ character and temperament.	2	2	**0	2
51	Personality traits of patients.	1	-2	2	-2
52	Aggressive behavior exhibited by patients.	0	4	0	0

* Asterisk (*) Indicates Significance at *P* < 0.05; Asterisk (**) Indicates Significance at *P* < 0.01)

### Factor 1: organizational factors and work conditions

Factor 1, which accounted for 20% of the total variance, was represented by the perspectives of 19 nurses. The items included in this factor were excessive workload (**5), low salaries received by nurses (**5), inadequate provision of comfort facilities for nurses (*4), job dissatisfaction among nurses (4) and a high number of shifts (4). Some illustrative quotes that exemplify Factor 1:

“*We’re constantly moving from one patient to another, with scarce time to communicate or explain things clearly. The pace is overwhelming and often leads to misunderstandings.”* (Nurse Participant).

*“Constant stress and overwork make it challenging to maintain patience and empathy with patients. Job dissatisfaction adversely affects our communication.”* (Nurse Participant).

*“Frankly, the low salaries hinder our sense of value within the hospital administration. This affects morale and ultimately influences our interactions with patients.”* (Nurse Participant).

*“We scarcely have time for a proper lunch break, much less a moment to relax and recharge. The absence of basic amenities generates significant tension and frustration, which can negatively affect our interactions with patients.”* (Nurse Participant).

*Working extended hours and multiple shifts drains our energy and affects our overall well-being. This can compromise our communication skills and patience with patients.”* (Nurse Participant).

### **Factor 2**: **emotional distress and psychological barriers**

Factor 2: Seven participants loaded significantly on factor 2, which was explained as 11% of the total variance. The items consisting of this factor were inappropriate behavior of patients’ companions (**5), patients asking unnecessary questions (**5), a high number of shifts (4), aggressive behavior exhibited by patients (4), and anxiety, pain, and physical discomfort experienced by patients (**4). Some illustrative quotes that exemplify Factor 2:

*“Sometimes patients are in a lot of pain or feeling very anxious, which can make communication difficult. They might struggle to express themselves clearly or become easily frustrated.”* (Nurse Participant).

*“Dealing with demanding or aggressive family members can be very stressful. It can be hard to focus on communicating effectively with the patient when their companions are causing disruptions.”* (Nurse Participant).

*“While I understand patients want to be informed, sometimes the constant barrage of questions, especially when I’m already overloaded with work, can be overwhelming and make it difficult to provide clear and concise explanations.”* (Nurse Participant).

*“Working long hours and multiple shifts can lead to burnout and emotional exhaustion. This can make it harder to manage difficult situations with patients and communicate with them patiently.”* (Nurse Participant).

### Factor 3: lack of mutual understanding and awareness

Four nurses loaded on factor 3 accounted for 7% of the total variance. The items incorporated in this factor were the patient’s lack of awareness regarding the role and responsibilities of nurses (5), a high number of shifts (5), a low level of awareness among patients (4), nurses’ limited awareness of the concept of communication and communication skills (**4), and insufficient training on communication principles provided to nurses (4). Some illustrative quotes that exemplify Factor 3:

*“Some patients have unrealistic expectations of what nurses can do. They might not understand the limitations of our role and get frustrated when we cannot fulfill all their requests. This can lead to misunderstandings and communication breakdowns.”* (Nurse Participant).

*“There seems to be a general lack of awareness from some patients about healthcare procedures and their own health conditions. This can make it difficult to explain things clearly and ensure they understand the information being provided.”* (Nurse Participant).

*“While we focus on medical knowledge and patient care, there hasn’t been much emphasis on communication skills development. Sometimes, I struggle to explain things in a way that’s easy for patients to understand.”* (Nurse Participant).

*“We haven’t received specific training on effective communication techniques for dealing with patients from diverse backgrounds or those experiencing emotional distress. This can lead to missed cues and misunderstandings.”* (Nurse Participant).

### Factor 4: declining professional motivation and engagement among nurses

Seven study participants loaded significantly on factor 4, which explained 9% of the total variance. The items that consisted of this factor were the patient’s lack of awareness regarding the role and responsibilities of nurses (5), lack of interest and motivation among nurses towards their profession (5), job dissatisfaction among nurses (4), inadequate supervision of nurses (4), and absence of feedback from patients (4). Some illustrative quotes that exemplify Factor 4:

*“Feeling undervalued and not receiving any feedback on our work can be disheartening. It takes a toll on motivation and makes it harder to find passion in the job, which can ultimately affect communication with patients.* (Nurse Participant)

*“When you’re constantly stressed, overworked, and undervalued, it’s easy to lose interest and motivation in your profession. This can lead to a more detached approach to communication with patients.”* (Nurse Participant).

*“The lack of proper supervision and support can make us feel overwhelmed and unsure of ourselves. This can lead to decreased confidence and motivation, impacting our communication skills and interactions with patients.”* (Nurse Participant).

## Discussion

The purpose of this study was to identify communication barriers between nurses and patients from the perspective of Iranian nurses using Q methodology. In this study, four factors including organizational factors and work conditions (20%), emotional distress and psychological barriers (11%), lack of mutual understanding and awareness (7%), and declining professional motivation and engagement among nurses (9%). were identified and explained 47% of the variance. Our study showed that excessive workload was the main barrier for communication barriers between nurses and patients. In line with this finding, a previous study showed that excessive workload has been identified as a prominent factor affecting nurse-patient communication [6, 21]. Nurses often face time constraints and competing responsibilities, leaving limited time for meaningful patient interactions. This can hinder the establishment of rapport, active listening, and effective information exchange. A study by Havaei and Maura (2020) found that nurses’ heavy workloads were associated with lower patient satisfaction scores, indicating the impact of workload on communication quality [22].

Low salaries received by nurses can lead to job dissatisfaction, which can indirectly affect nurse-patient communication. Studies have shown that lower job satisfaction is associated with decreased motivation and engagement in patient care [23, 24]. When nurses are dissatisfied with their compensation, it can impact their overall job satisfaction, morale, and ultimately, their communication with patients.

Our study showed that inadequate provision of comfort facilities, such as rest areas or break rooms, can also contribute to communication barriers. Previous studies have shown that without appropriate spaces for nurses to recharge and relax during their shifts, they may experience increased stress and fatigue, which can impact their ability to communicate effectively with patients [25, 26]. The lack of comfortable and supportive work environments may hinder nurses’ well-being and, subsequently, their communication skills.

Job dissatisfaction among nurses is another organizational factor that can hinder effective communication. When nurses are dissatisfied with their work conditions, it can lead to decreased job engagement, increased turnover rates, and a negative work environment [27, 28]. This negative atmosphere can affect communication and collaboration with patients, potentially leading to suboptimal care experiences.

A high number of shifts worked by nurses can contribute to communication barriers due to fatigue and burnout. Fatigue can impair cognitive functioning and communication skills, hindering effective information exchange and understanding of patients’ needs [29]. The demanding schedules and long working hours can limit nurses’ energy and attentiveness during patient interactions, potentially leading to miscommunication or misunderstandings.

When patients are experiencing emotional distress or psychological challenges, they may find it difficult to effectively express their needs, concerns, or comprehend the information provided by nurses. This can result in miscommunication, misunderstandings, and hindered information exchange [30]. This finding is consistent with the results of our study. Patients’ lack of awareness regarding the role and responsibilities of nurses can further exacerbate these challenges, as they may have unrealistic expectations or misunderstand the scope of nursing practice [31].

According to the present results, nurses’ limited awareness of communication concepts and skills, as well as insufficient training on communication principles, can hinder effective communication with patients [6]. Without a solid understanding of communication techniques, active listening, and patient-centered care, nurses may struggle to establish rapport, address patients’ emotional needs, and convey information in a clear and compassionate manner.

The impact of emotional distress and psychological barriers on nurse-patient communication has been recognized in the literature. A study by Beck, Dracup, and Hamilton (2006) found that patients experiencing emotional distress, such as anxiety or depression, had difficulty communicating their symptoms and needs to healthcare providers [32]. Previous studies have highlighted the crucial role of emotional cues in patient-centered communication, emphasizing the importance of healthcare providers’ recognition and response to patients’ emotional states [33, 34].

The three challenge extracted in this research were lack of mutual understanding and awareness. When patients are unaware of the specific roles and responsibilities of nurses, they may have unrealistic expectations or misunderstand the scope of nursing practice. This can lead to miscommunication and frustration on both sides. Patients may not fully understand the expertise and limitations of nurses, which can hinder effective communication and collaboration [9, 35].

On the other hand, if patients have a low level of awareness regarding their healthcare condition or treatment process, they may struggle to effectively communicate their needs or understand the information provided by nurses. Limited health literacy or lack of access to education can contribute to this lack of awareness, hindering effective communication and shared decision-making [36, 37]. A study by Street, Makoul, Arora, and Epstein (2009) highlighted the importance of patient-centered communication, emphasizing the need for healthcare providers to elicit patients’ perspectives, address their concerns, and provide information in a way that aligns with their understanding and preferences [38].

The communication barriers resulting from declining professional motivation and engagement among nurses have been recognized in research. A study by Laschinger et al. (2014) found that nurse burnout, which is closely linked to motivation and engagement, negatively affected nurse-patient communication and patient satisfaction [39]. Another study by Van Bogaert et al. (2014) highlighted the impact of nurse work engagement on patient-centered care, emphasizing the importance of fostering a positive work environment to enhance nurse-patient communication [40]. Nurse-patient communication is a vital component of quality healthcare delivery. Nurses who are motivated and engaged in their profession are more likely to exhibit effective communication skills, actively listen to patients, and provide empathetic care. However, when nurses experience a decline in professional motivation, they may become disengaged, leading to various communication barriers.

## Conclusion

In conclusion, several factors contribute to the communication barriers between nurses and patients. These include an excessive workload, low salaries, inadequate comfort facilities, fatigue, patients’ emotional distress, a lack of awareness, and limited communication skills among nurses. To address these barriers, it is necessary to manage the workload, improve job satisfaction, provide supportive environments, address patients’ emotional needs, enhance patient education, and offer communication training for nurses. By addressing these factors, healthcare organizations can promote effective communication and thereby enhance the quality of patient care.

## Data Availability

Data sets generated during the current study are available from the corresponding author on reasonable request.

## Abbreviations

IOM: Institute of Medicine
MEBoT: Medical Ethics Board of Trustees
TUMS: Tehran University of Medical Sciences
